# Electromechanical and structural phenotypes from cardiac imaging and epicardial mapping in inherited arrhythmia syndromes

**DOI:** 10.3389/fcvm.2026.1900085

**Published:** 2026-07-22

**Authors:** Eunae Sandra Cho, Seung-Young Roh

**Affiliations:** 1 Department of Oral Pathology, Oral Cancer Research Institute, Yonsei University College of Dentistry, Seoul, Republic of Korea; 2 BK21 FOUR Project, Yonsei University College of Dentistry, Seoul, Republic of Korea; 3 Division of Cardiology, Department of Internal Medicine, Korea University College of Medicine and Korea University Medicine, Seoul, Republic of Korea

**Keywords:** Brugada syndrome, cardiac magnetic resonance imaging, epicardial mapping, genetic testing, inherited arrhythmia syndromes, long QT syndrome

## Abstract

Inherited arrhythmia syndromes—including Brugada syndrome (BrS), long QT syndrome (LQTS), catecholaminergic polymorphic ventricular tachycardia (CPVT), and short QT syndrome (SQTS)—can cause malignant arrhythmias and sudden cardiac death without overt structural heart disease on conventional evaluation. Advances in cardiac magnetic resonance imaging (CMR), speckle-tracking strain echocardiography, and epicardial electroanatomical mapping have revealed previously unrecognized electromechanical and structural abnormalities in these conditions. In BrS, right ventricular outflow tract (RVOT) fibrosis, connexin-43 downregulation, and progressive structural remodeling support the concept of a concealed subepicardial substrate. In LQTS, prolonged contraction duration, mechanical dispersion, and diastolic dysfunction represent an electromechanical phenotype beyond QT prolongation, and preliminary evidence of a right ventricular epicardial substrate has been reported. CPVT and SQTS largely preserve the classical electrical disease model, though limited CMR data suggest possible structural overlap in specific CPVT genotypes. This review summarizes disease-specific findings from advanced imaging, considers the role of genetic testing in guiding phenotypic assessment, and discusses the clinical implications of these evolving concepts.

## 1 Introduction

Inherited arrhythmia syndromes are an important cause of sudden cardiac death (SCD), accounting for an estimated 5%–10% of cases, particularly in young individuals without overt structural heart disease (1). Brugada syndrome (BrS) has a global prevalence of approximately 0.05% and long QT syndrome (LQTS) affects roughly 1 in 2,000 (2, 3), with substantial psychological and economic burdens on families through hereditary risk, ICD therapy, and lifelong follow-up (1).

These syndromes share a common framework: genetically determined abnormalities in cardiomyocyte ion channels and regulatory proteins cause malignant arrhythmias in the absence of overt structural heart disease on standard assessment. Current ESC, AHA, and HRS guidelines classify them separately from structural heart diseases (4). Three decades of genetic research have identified causative variants in many genes, although systematic gene–disease curation through ClinGen has refined the list of clinically actionable genes and shown that many previously proposed candidates lack sufficient evidence for clinical use (5–8). Genetic testing has an established role in diagnostic confirmation and family cascade screening, but its direct impact on prognosis remains limited (5).

Recent advances in imaging have broadened this framework. CMR with late gadolinium enhancement (LGE) and parametric mapping, speckle-tracking strain echocardiography, and percutaneous epicardial electroanatomical mapping (EAM) have uncovered substrates not apparent on conventional assessment. In BrS, RVOT epicardial fibrosis and connexin-43 downregulation have been identified; in LQTS, strain echocardiography has revealed an electromechanical phenotype that extends beyond QT prolongation (9–14).

This review outlines advances in cardiac imaging and EAM (Section 2), provides a disease-specific assessment across BrS, LQTS, CPVT, SQTS, and *SCN5A* overlap syndromes (Section 3), and discusses the role of genetic testing (Section 4), discussion (Section 5), and conclusion (Section 6).

## 2 Advances in cardiac imaging and mapping

### 2.1 Advanced cardiac imaging

Cardiac imaging has advanced markedly over the past two decades, enabling noninvasive detection of myocardial changes that previously required biopsy or autopsy for identification. CMR is the most powerful noninvasive tool for myocardial tissue characterization. LGE detects focal fibrosis and scarring with high spatial resolution; T1 mapping and extracellular volume fraction (ECV) measurement allow quantification of diffuse interstitial fibrosis; T2 mapping identifies myocardial edema and inflammation; and cine CMR provides precise assessment of biventricular volume, function, and regional wall motion (15).

Speckle-tracking strain echocardiography quantifies segmental myocardial deformation. Global longitudinal strain (GLS) is a sensitive marker of left ventricular systolic function, while measurement of time-to-peak strain in each segment allows quantification of contraction duration and its segmental variability, termed mechanical dispersion. These parameters can detect subtle abnormalities in patients with normal ejection fraction, chamber size, and wall motion on conventional echocardiography. This is particularly valuable in inherited arrhythmia syndromes, in which gross structure is typically preserved but subtle functional and indirect structural abnormalities may already be present.

### 2.2 Epicardial electroanatomical mapping and ablation

Percutaneous epicardial access, first described by Sosa et al. in 1996, enables direct assessment of epicardial myocardial electrical properties in living patients (16). High-density EAM records hundreds to thousands of electrograms, allowing detailed analysis of local voltage, electrogram morphology, and conduction velocity. Normal myocardium shows uniform voltage and smooth waveforms, whereas areas of fibrosis or fatty infiltration exhibit low voltage, fractionated electrograms, and conduction delay—abnormalities that can be detected even when standard echocardiography and CMR appear normal. In BrS, radiofrequency ablation of abnormal epicardial regions identified by EAM has been associated with normalization of the ECG phenotype and reduction in VF recurrence, indirectly supporting the clinical significance of these substrates.

## 3 Disease-specific assessment

### 3.1 Brugada syndrome: concealed structural substrate

BrS, first described in 1992, was historically viewed as a primary electrical disorder, with loss-of-function *SCN5A* variants identified in a minority of cases. Arrhythmogenesis was attributed to repolarization or depolarization/conduction abnormalities in the RVOT, both hypotheses assuming a structurally normal heart (3, 17). Recent studies using histopathology, imaging, and epicardial mapping have substantially expanded this framework.

#### 3.1.1 Cardiac MRI findings

Noninvasive imaging has provided evidence of structural changes in living BrS patients. A systematic review by De Raffele et al. found increased RVOT volumes and regional wall motion abnormalities (RWMA) in approximately 31% of patients (9). Gray et al. found abnormal RVOT morphology in 67%, with rare genetic variants present exclusively in those with morphological abnormalities (36% vs. 0%, *p* = .02)—suggesting that structural changes may develop in an age- and gene-dependent manner (18).

Isbister et al. extended these findings in a longitudinal study (19): among 18 asymptomatic BrS patients with initially normal CMR, repeat imaging at a mean of 5 years revealed new focal septal LGE in 22% (4/18). This was the first longitudinal demonstration that structural changes in BrS can progress over time, suggesting that BrS may involve an evolving structural process rather than a fixed electrical abnormality.

#### 3.1.2 Histopathological evidence

Autopsy and explanted heart studies have shown interstitial fibrosis, fibrofatty replacement, and inflammatory infiltrates in the RVOT epicardium of BrS patients. Nademanee et al. established the link between these findings and the electrophysiological abnormalities, demonstrating significant interstitial fibrosis and reduced connexin-43 expression in the RVOT epicardium (10)—a pathological basis for the fractionated electrograms, low voltage, and conduction delay observed on EAM.

#### 3.1.3 Epicardial substrate and ablation

Epicardial mapping has clarified the relationship between the surface ECG phenotype and the arrhythmogenic substrate. In a series of studies, the extent of the abnormal RVOT epicardial substrate was closely correlated with both the presence and amplitude of the spontaneous type 1 ECG pattern, and sodium-channel blocker challenge unmasked additional substrate in proportion to the degree of coved-type ST-segment elevation (20–22). Substrate size was also shown to have independent prognostic significance. These findings suggest that the type 1 ECG is not only a diagnostic marker but also a surface manifestation of the underlying substrate burden.

The BRAVO (Brugada Ablation of VF Substrate Ongoing) multicenter registry reported outcomes of epicardial radiofrequency ablation in 159 high-risk BrS patients (all with spontaneous VF, 80% recurrent), with significant reduction in VF recurrence and normalization of pathologic ST elevation in most. The subsequent BRAVE randomized trial extended this from observational to controlled-trial evidence, supporting substrate-based ablation and reinforcing the concept that structural abnormalities play a functional role in arrhythmogenesis (11, 23).

Left ventricular involvement has also been reported. Cheniti et al. found epicardial substrate abnormalities involving the left ventricle in 10 of 22 extensively mapped BrS patients (24); these patients had higher rates of pathogenic *SCN5A* variants and longer arrhythmia histories, suggesting that structural involvement may increase with disease duration and genetic burden.

#### 3.1.4 Subepicardial cardiomyopathy and relationship with ARVC

Miles et al. synthesized these converging lines of evidence to propose that BrS, early repolarization syndrome (ERS), and idiopathic VF may share a common substrate of subtle microstructural abnormalities in the right ventricular subepicardium—a “subepicardial cardiomyopathy” (12).

This concept raises the question of how BrS relates to arrhythmogenic right ventricular cardiomyopathy (ARVC), an overt, progressive cardiomyopathy caused primarily by desmosomal gene mutations and producing global RV dysfunction with fibrofatty replacement. Although BrS shares some features with ARVC—RV involvement, epicardial abnormalities, right precordial ECG changes, and comparable RVOT volumes (18)—the structural changes in BrS remain subtle, largely confined to the RVOT, and accompanied by preserved RV function. BrS is therefore best characterized not as an overt cardiomyopathy but as an inherited arrhythmia syndrome with a concealed structural substrate, with the boundary between the two expected to blur as imaging resolution improves (12).

### 3.2 Long QT syndrome: the electromechanical phenotype

LQTS is characterized by QT interval prolongation and susceptibility to torsades de pointes. Unlike BrS, the new findings in LQTS center not on overt fibrosis but on the mechanical consequences of ion channel dysfunction—an electromechanical phenotype that extends beyond QT prolongation (13, 25).

#### 3.2.1 The mechanical meaning of QTc prolongation

QTc prolongation reflects abnormally prolonged action potential duration (APD) in cardiomyocytes, which lengthens contraction. Because APD prolongs to different degrees in different myocardial layers (endocardium, midwall, epicardium) and regions (base, mid, apex), this heterogeneity creates segmental differences in contraction onset and offset—manifesting as mechanical dispersion. QTc prolongation therefore reflects not only an electrical abnormality but also spatial and temporal heterogeneity in myocardial contraction.

#### 3.2.2 Mechanical dispersion and contraction abnormalities

The link between LQTS and mechanical dysfunction was first noted approximately 30 years ago with M-mode echocardiography, which revealed prolonged left ventricular contraction and a double-peaked contraction pattern associated with arrhythmic events (26). Tissue Doppler imaging (TDI) and speckle-tracking echocardiography (STE) later enabled systematic quantification (13).

Haugaa et al. demonstrated that LQTS mutation carriers had significantly prolonged contraction duration compared with controls (445 ± 45 vs. 390 ± 40 ms, *p* < .001), and this prolongation persisted (≥0.44 s) in symptomatic carriers not identified by QTc—suggesting that echocardiography can identify high-risk individuals missed by ECG (13). The same group later demonstrated transmural mechanical dispersion, with longer longitudinal than circumferential contraction duration (460 ± 45 vs. 445 ± 45 ms, *p* = .008) (27).

In the largest genotyped cohort, Leren et al. studied 192 LQTS patients (139 LQT1, 53 LQT2) and found reduced GLS, prolonged contraction duration, and increased mechanical dispersion, with genotype-specific patterns. Diastolic dysfunction (reduced e’, 10.7 ± 2.7 vs. 12.5 ± 2.0 cm/s) and left atrial enlargement (∼20%) were also observed. A subsequent meta-analysis confirmed that left ventricular contraction duration was the strongest predictor of cardiac events in LQTS, outperforming QTc (25, 28).

A complementary parameter is the electromechanical window (EMW)—the difference between the end of electrical systole (QT end) and the end of mechanical systole (aortic valve closure). EMW negativity, indicating that electrical systole outlasts mechanical systole, can identify symptomatic LQTS patients and may complement or even outperform QTc in risk stratification (29, 30).

#### 3.2.3 Epicardial substrate: a preliminary finding

Pappone et al. performed high-density epicardial EAM in 11 high-risk LQTS patients with recurrent ICD shocks for VF and identified low-voltage areas, fractionated electrograms, and conduction delay localized to the right ventricular epicardium; the left ventricular endocardium and epicardium were unremarkable. Radiofrequency ablation of these abnormal regions suppressed VF recurrence (14).

The study was limited by small sample size and lack of genotype-specific analysis, and an accompanying editorial noted that the findings require confirmation in larger cohorts (31). Nevertheless, it raises the possibility that some high-risk LQTS patients harbor a subclinical substrate analogous to that in BrS.

#### 3.2.4 From functional abnormality to structural remodeling

The electromechanical abnormalities in LQTS are direct functional consequences of ion channel dysfunction. Whether long-standing heterogeneous contraction eventually generates focal mechanical stress contributing to secondary remodeling or microfibrosis remains unknown and warrants longitudinal study (19).

### 3.3 Catecholaminergic polymorphic ventricular tachycardia

CPVT is characterized by normal resting ECG, absence of structural heart disease on standard imaging, and exercise- or stress-triggered bidirectional or polymorphic ventricular tachycardia, caused by aberrant diastolic calcium release from the sarcoplasmic reticulum due to mutations in *RYR2* (autosomal dominant) or *CASQ2* (autosomal recessive)—a primarily electrical mechanism (1, 5, 32).

A multicenter CMR study of 20 genetically confirmed CPVT patients with normal echocardiograms found that all three carriers of *RYR2* exon 3 deletions had structural abnormalities, including prominent trabeculation suggestive of left ventricular noncompaction (LVNC) and abnormal RV relaxation; one *RYR2* missense carrier also met LVNC criteria (33). Whether aberrant calcium release can directly affect myocardial structure is biologically plausible but unconfirmed.

Overall, CPVT remains the inherited arrhythmia syndrome in which the classical electrical model is most applicable. Genotype-specific structural overlap is currently restricted to a small number of cases and not practice-changing; whether long-term follow-up will reveal additional structural evolution remains to be determined.

### 3.4 Short QT syndrome

SQTS is the rarest inherited arrhythmia syndrome, and evidence on structural or mechanical phenotypes is very limited. The 2022 international expert consensus recommended routine genetic testing for *KCNH2* and *KCNQ1* in patients with high-probability SQTS, with *KCNJ2* and *SLC4A3* considered only in selected cases. Current ClinGen gene-validity curation, however, classifies only *KCNH2* as having definitive evidence for SQTS, with *KCNQ1*, *KCNJ2*, and *SLC4A3* assigned lower levels (5, 8). No systematic structural assessment has been performed in SQTS. We therefore classify SQTS as a condition where the classical electrical model is maintained, while noting that this reflects data scarcity rather than confirmed absence of structural substrates.

### 3.5 *SCN5A* overlap syndromes and inherited sinus node disease

*SCN5A* variants have been implicated in BrS, LQT3, dilated cardiomyopathy (DCM), sick sinus syndrome, and atrial arrhythmias, and different phenotypes can manifest within the same family or in the same patient at different life stages. *SCN5A* overlap syndromes exemplify how an identical genetic lesion can present as a purely electrical disorder in one setting and as overt cardiomyopathy in another (34).

Inherited sinus node dysfunction (SND) further illustrates this convergence. Although most SND results from age-related degenerative fibrosis and is not genetically determined, variants in ion channel genes (*SCN5A*, *HCN4*) and structural protein genes (*MYH6*, *TTN*, *LMNA*) have been associated with SND, and a recent GWAS identified the *KRT8* locus—entirely unrelated to ion channels—as a susceptibility factor (35, 36). These observations show that electrical and structural pathways can converge to produce clinical disease.

**Figure 1:**
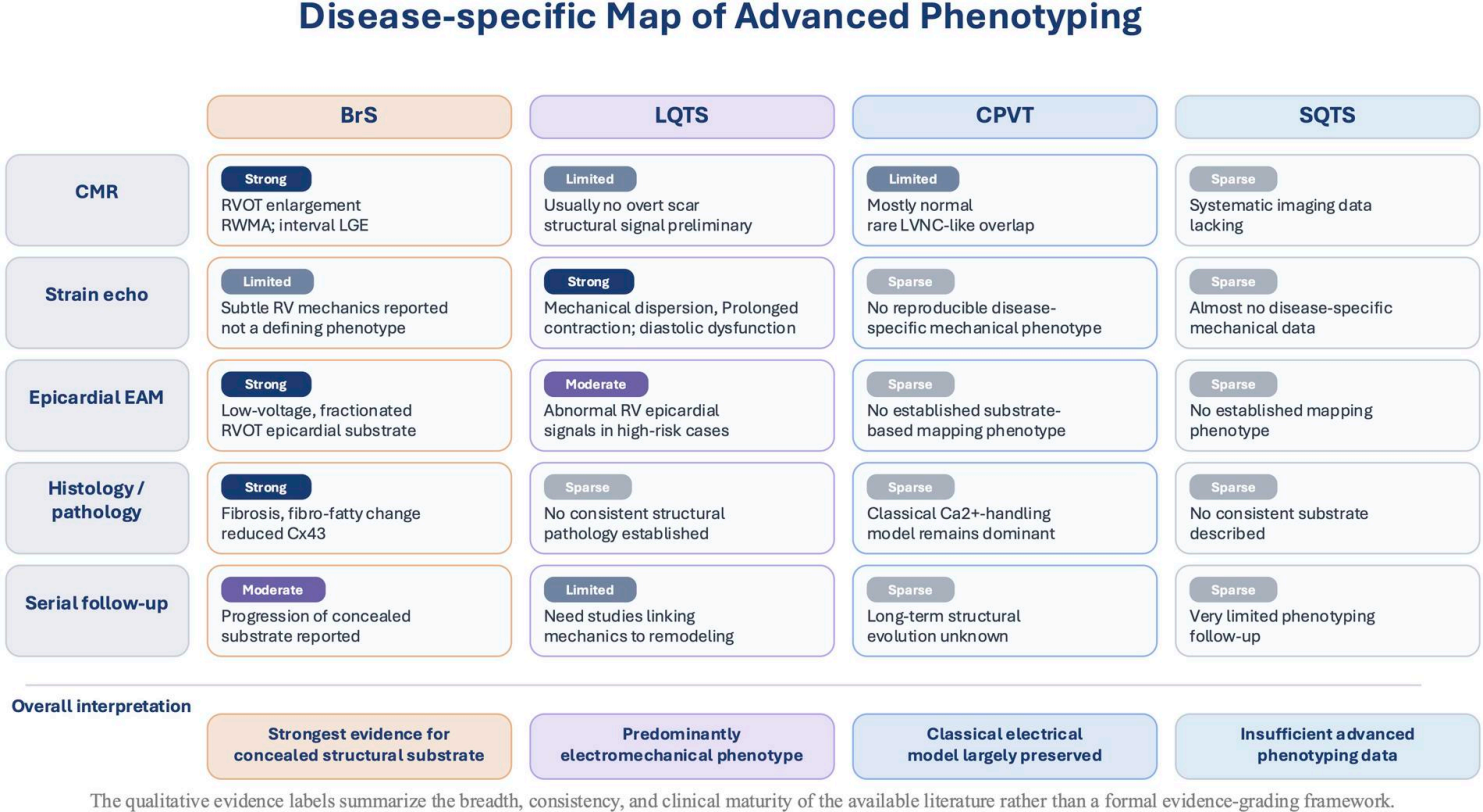
Disease-specific map of advanced phenotyping. Matrix comparing the maturity and direction of current evidence for structural and electromechanical substrates across BrS, LQTS, CPVT, and SQTS. Five phenotyping domains are evaluated: CMR, strain echocardiography, epicardial EAM, histology/pathology, and serial follow-up. Evidence strength for each disease–modality combination is graded qualitatively (strong, moderate, limited, or sparse) to summarize the breadth, consistency, and clinical maturity of the available literature rather than a formal evidence-grading framework. Bottom row provides an overall interpretation for each syndrome.

Together, these examples suggest that patients initially diagnosed with an inherited arrhythmia syndrome who later develop conduction disease, atrial fibrillation, or systolic dysfunction may benefit from reassessment over time (34). Table 1 summarizes the maturity and direction of current evidence across imaging, mapping, and histopathological domains, and Figure 1 uses a qualitative grading scheme across five phenotyping domains to enable rapid comparison between syndromes.

**Table 1:** Disease-Specific reassessment of inherited arrhythmia syndromes by advanced phenotyping.

Disease	Key findings from advanced assessment	Nature of substrate	Progression/follow-up
BrS	CMR: RVOT enlargement (67%), RWMA (31%), progressive LGE (22% over 5 yrs) Histology: interstitial fibrosis, fibrofatty change, reduced Cx43 EAM: RVOT epicardial low voltage and fractionated EGM; LV involvement in ∼50% of extensively mapped patients Ablation: ECG normalization and reduced VF recurrence	Concealed RVOT-predominant substrate May progress over time	Progressive phenotype New LGE after 5 yrs Structural extent correlates with arrhythmia duration and SCN5A status
LQTS	Strain echo: prolonged contraction (445 vs. 390 ms), increased mechanical dispersion (SD of time-to-peak strain: 33 vs. 21 ms), transmural dispersion, diastolic dysfunction, negative EMW, genotype-specific patterns EAM: RV epicardial low voltage and fractionated EGM in high-risk patients (*n* = 11; preliminary) Ablation: VF suppression reported	Electromechanical phenotype Epicardial substrate remains preliminary	Longitudinal studies needed to determine whether sustained electromechanical dysfunction leads to secondary structural remodeling
CPVT	CMR: LVNC-like overlap in RYR2 exon 3 deletions (3/3); RV relaxation abnormalities in selected cases Overall: no established mechanical or mapping phenotype	Limited evidence Restricted to specific RYR2 variants	Structural evolution unknown Current data do not support routine serial advanced imaging Targeted reassessment may be considered in selected genotypes or evolving phenotypes
SQTS	Current evidence: no systematic imaging or mapping data available	Not established	Very limited phenotyping and follow-up data Reflects data scarcity rather than confirmed absence
SCN5A overlap	Spectrum: from normal imaging to overt DCM BrS phenotype: epicardial substrate, more extensive with longer arrhythmia history	Entire spectrum ranges from isolated electrical dysfunction (BrS phenotype) to overt dilated cardiomyopathy	Life-course evolution possible Same patient may shift from electrical to structural phenotype

BrS, Brugada syndrome; CMR, cardiac magnetic resonance; CPVT, catecholaminergic polymorphic ventricular tachycardia; Cx43, connexin-43; DCM, dilated cardiomyopathy; EAM, electroanatomical mapping; ECG, electrocardiogram; EGM, electrogram; EMW, electromechanical window; LGE, late gadolinium enhancement; LQTS, long QT syndrome; LV, left ventricle; LVNC, left ventricular noncompaction; RV, right ventricle; RVOT, right ventricular outflow tract; RWMA, regional wall motion abnormality; SQTS, short QT syndrome; VF, ventricular fibrillation.

## 4 Advances and role of genetic testing

Genetic testing has advanced markedly in the evaluation of inherited arrhythmia syndromes. Next-generation sequencing and ClinGen-based gene curation have made molecular diagnosis possible at an unprecedented level. Diagnostic yields are approximately 75% in clinically confirmed LQTS (7), 60% in classical CPVT (8), and 20%–30% in BrS (*SCN5A* only) (6). The strongest established role of genetic testing is diagnostic refinement in probands and predictive cascade screening in first-degree relatives, identifying at-risk family members before symptoms develop. Although routine testing of all patients with asymptomatic ECG abnormalities is not practical, it should be considered in patients with a family history or clinical suspicion of inherited arrhythmia (5).

The expansion of genetic testing is directly linked to the discovery of structural and electromechanical phenotypes. Although routine advanced imaging for all inherited arrhythmia patients is not feasible, genotype information can help prioritize patients most likely to benefit. The observations that *SCN5A*-positive BrS patients have more frequent RVOT abnormalities and LV involvement, and that LQT1 and LQT2 show different mechanical patterns, illustrate how genotype data can guide targeted imaging (18, 24, 25). Genetic testing is thus evolving from a diagnostic tool toward a means of guiding phenotypic reassessment and individualized risk stratification.

## 5 Discussion

### 5.1 Expanding the concept of inherited arrhythmia syndromes

The evidence reviewed here suggests that the diagnosis of an inherited arrhythmia syndrome should not preclude concurrent structural or electromechanical abnormalities. These conditions are better understood as a spectrum—from electrical to mechanical to subtle structural changes—where BrS, LQTS, ARVC, and DCM are not always sharply distinct. *SCN5A* overlap syndromes best illustrate this, as the same genetic lesion can produce a purely electrical phenotype at one stage and structural cardiomyopathy at another (12, 34).

### 5.2 Expanding the role of advanced imaging

The 12-lead ECG remains the diagnostic cornerstone of inherited arrhythmia syndromes. In BrS, the dynamic type 1 pattern may also reflect epicardial substrate expression. However, a single conventional ECG and routine echocardiography alone cannot fully capture the evolving phenotype of these conditions. Serial standardized ECG assessment, strain echocardiography (mechanical dispersion, contraction duration), and CMR (RVOT morphology, tissue characterization, LGE) provide complementary phenotypic and diagnostic information (9, 13, 25).

A key point is the value of serial assessment. As demonstrated by Isbister et al. in BrS, structural abnormalities may not be evident at initial evaluation and become apparent only years later (19), supporting longitudinal reassessment in selected patients—though not routine serial imaging for all. As newer modalities such as cardiac diffusion tensor imaging, 4D flow MRI, and high-resolution parametric mapping become available, additional findings are likely to emerge, with growing roles in risk stratification and treatment planning.

### 5.3 Future perspectives

Future work should apply emerging imaging techniques to large multicenter cohorts of BrS and LQTS patients to establish the prevalence and prognostic significance of concealed substrates, and clarify the mechanisms by which *SCN5A* and related genes drive both electrical dysfunction and structural change. Integration of genetic and imaging data for individualized risk assessment will be a key direction (5, 7, 34).

## 6 Conclusion

In BrS, converging evidence from CMR, histopathology, and EAM supports a concealed RVOT-predominant substrate, and epicardial ablation targeting this substrate has opened a new therapeutic avenue. In LQTS, electromechanical phenotypes beyond QT prolongation carry independent prognostic value. Inherited arrhythmia syndromes are not defined solely by arrhythmias, nor should their evaluation be limited by the assumption that structural abnormalities are absent.
